# The Uses and Limitations of Surgery

**Published:** 1905-04-15

**Authors:** W. Essex Wynter

**Affiliations:** Physician and Lecturer on Medicine to the Middlesex Hospital


### Hospital Clinics.

### THE USES AND LIMITATIONS OF SURGERY.

By W. Essex Wynter, M.D., F.R.C.P., Physician and Lecturer on Medicine to the Middlesex Hospital.

The most striking feature in the healing art in the last quarter of a century is the large share which surgery has claimed in the treatment of maladies formerly regarded as within the province of the physician; though it must be remembered that this included not only inflammatory, functional, or diffuse internal lesions, but such organic troubles of the viscera as were then confessedly beyond reach of the surgeon's art.

The more careful and intelligent observation of the results of injuries, especially in campaigns, and the application of surgical methods to exposed and injured viscera in attempts to imitate or hasten natural processes of recovery, naturally led up to more systematic and deliberate surgical interference in visceral disease.

With the advent of anæsthesia and asepsis the domain of surgery rapidly extended, and it is not surprising that with the simultaneous development of pathology, associated with systematic post-mortem examination, enormous advances have been made in the removal or remedy of local causes of disease. With increasing success these have been more and more venturesome, till there is now scarcely any organ or part of the body that has not been the subject of surgical interference.

There is, moreover, an increasing tendency for surgeons to deal with such cases at first hand without, as formerly, being guided or referred to by the physician. It cannot be denied that the necessity for satisfying himself of the probable nature of a lesion and the feasibility of remedy has led the surgeon to an accuracy in gauging the nature and limits of local disease rivalling that of his medical confrère; while the sense of keen personal responsibility in the undertaking, and the familiarity with morbid changes in all their aspects acquired in the course of operations, give the surgeon peculiar advantages.

The fame of many successes has induced a tide in the direction of operation, encouraged not only by medical men but by patients themselves who seek radical cures and the immediate relief which surgery frequently and even exclusively affords.

There is, however, another aspect to this matter. Apart from the individual factor there are risks, remote for the most part, in even the smallest operation which cannot be absolutely eliminated by the most skilful management. Hæmorrhage, sepsis, and failure of the vital functions under anæsthetics still claim a certain proportion of victims, and even when an operation has been satisfactorily completed immediate and permanent relief of the condition for which it was performed does not invariably result.

Failure in such cases is not to be attributed to the manner in which the operation was carried out, but in many instances to inadequate knowledge beforehand, and the indiscriminate application of some special operative procedure to a variety of conditions anatomically or pathologically different. It is satisfactory to observe that notwithstanding the success that has attended the development of his art in recent years, the surgeon is becoming more critical as to the need, prospect, and condition of his patient before committing himself to the responsibility of an operation.

Where the patient is already in the care of a physician it is often with some anxiety and misgiving that surgical aid is invoked. In his own practice the physician is controlled by the maxim that if he can do no good he will at least do no harm with the measures he employs, but with surgery he is bound to recognise that limits cannot be put to the effects of interference, which may pass beyond the control of himself or the operator. Setting aside those comparatively rare accidents, which mar the results of the most perfected operation, by introducing a mortality rate of about two per cent., and which can only be taken into account as a restraining influence in lightly recommending operation in conditions not unbearable or beyond the reach of other remedies, what are the factors which lead to untoward results?

First there is erroneous diagnosis either of local disorder where none exists, or a faulty appreciation of actual lesion, which may not when brought into view be amenable to direct or even indirect treatment, or in more favourable cases may involve an incision in an unsuitable situation, or a consultation and change of tactics after the commencement of the operation.

Secondly, there is the overlooking or underestimation of important constitutional conditions in the patient which greatly increase the risk, and would sometimes forbid the performance of an operation, or considerably modify its extent. Such conditions as chronic heart or kidney disease, diabetes, tubercle, and Addison's disease, and the exhausting effect of long illness are readily recognised and taken into account, but there is greater difficulty in appreciating developmental defects and the results of degeneration and there are phases of Grave's disease not very obvious in themselves which exert a most detrimental influence.

In recent years many instances have been observed in which an unexpected fatal result at, or immediately after, operation, or a peculiar feebleness of resistance to disease or accident has been found associated with enlarged thyroid gland, in some the thymus persisting as well. In elderly and obese people the mere maintenance of the supine position for a prolonged period after operation tends to promote bronchitis and hypostatic pneumonia, through embarrassment of cardiac action, which may prove fatal; while in other and younger subjects thrombosis occurs, especially in association with anæmia from any cause, leading to delay and impairment of convalescence or to sudden death by pulmonary obstruction, while the supervention of septic pneumonia on operations connected with the mouth, nose or air passages is an only too frequent occurrence. Nice discrimination of cases before deciding to operate is evidently essential, and in acute disease little time may be available for decision, but any anomaly of symptoms or absence of usual indications should be allowed due weight, and suggest delay and closer investigation.

Acute pancreatitis (associated with pain in the upper zone of the abdomen and incomplete emptying of the colon), has been mistaken for intestinal obstruction, and treated by laparotomy without benefit except the establishment of a correct diagnosis; indications resembling those of appendix disease and perforation of the stomach or intestine have also led to precipitate operation, which necessarily proved abortive and only increased the patient's risk, while instances of peritonitis unassociated with visceral disease which have suffered laparotomy to their detriment are by no means uncommon.

On the other hand, careful preliminary examination and deliberation have led to the avoidance of operation in cases of general peritoneal adhesion, or entanglement of the bowel which have defied liberation even at a subsequent autopsy, and would necessarily have rendered any attempt at relief by operation futile.

In chronic disorders the plea for extended consideration before deciding on operation, especially in abdominal cases, is stronger. Though it is still early to attempt to estimate the full value and applicability of gastro-enterostomy it may safely be asserted that where symptoms are associated with neurosis in the more importunate patients and where actual dilatation of the stomach exists in connection with loss of teeth the operation should be avoided in favour of other treatment.

Numerous cases occur in women in early middle life where gastric ulcer associated with chlorosis has from neglect of treatment resulted in penetration of the stomach wall and adhesion to adjoining parts, giving rise to persistent anæmia and ill-health with varying gastric symptoms. So far the certain diagnosis and mode of treatment of this condition has not been settled; many manage to lead an ordinarily active life for years while others find the condition unendurable. When such is the case and the patient is unable to earn a livelihood, operation must be considered. There are, of course, instances enough in which chronic ulcer, pyloric stenosis (congenital or acquired) and new growth render gastro-enterostomy imperative and so far the immediate results of the operation have proved satisfactory.

Instances are, fortunately, not common in which the question of section of the stomach or intestine for severe hæmorrhage presents itself. Hæmorrhage is common enough in a minor or moderate degree, and recovery has taken place after syncope; moreover, some of the most profuse hæmorrhages are of such a nature as to defy direct surgical treatment. These considerations make one hesitate to suggest so formidable a remedy, and by that time it is often too late to interfere with any prospect of success. Where the local lesion has been recognised first, and hæmorrhage of a severe character supervenes, surgical interference may be attempted, and even then short-circuiting appears to offer a better prospect of success than direct treatment.

Nephropexy, having had a longer period of trial as a remedy for the symptoms associated with movable kidney, offers a better subject for judgment. It has the advantage of involving less risk to life than is associated with operations on other abdominal viscera, but has not proved uniformly successful in relieving symptoms, and careful analysis of these is necessary if the operation is to be resorted to with confidence. For the most part symptoms which may be attributed to a mechanical origin from dragging or twisting of the ureter and adjacent organs are more likely to be remedied than those of so-called nervous origin. In those instances in which mobility of the kidney is part of a neurosis, with wasting or general laxity of attachment of the abdominal organs (Glenard's disease), the operation is not satisfactory.

In any case in which an operation is contemplated, the duration of illness and condition of the patient are very important factors in coming to a decision, and may fully justify the surgeon in refusing to operate even when the pathological condition is fairly obvious.

---

